# The Impact of Transformational Leadership in the Nursing Work Environment and Patients’ Outcomes: A Systematic Review

**DOI:** 10.3390/nursrep13030108

**Published:** 2023-09-11

**Authors:** Line Miray Kazin Ystaas, Monica Nikitara, Savoula Ghobrial, Evangelos Latzourakis, Giannis Polychronis, Costas S. Constantinou

**Affiliations:** 1 Department of Health Sciences, School of Life and Health Sciences, University of Nicosia, Nicosia 1700, Cyprus; 2 Department of Basic and Clinical Sciences, Medical School, University of Nicosia, Nicosia 1700, Cyprus

**Keywords:** nursing, transformational leadership, work environment, systematic review

## Abstract

Background: With the increasingly demanding healthcare environment, patient safety issues are only becoming more complex. This urges nursing leaders to adapt and master effective leadership; particularly, transformational leadership (TFL) is shown to scientifically be the most successfully recognized leadership style in healthcare, focusing on relationship building while putting followers in power and emphasizing values and vision. Aim: To examine how transformational leadership affects nurses’ job environment and nursing care provided to the patients and patients’ outcomes. Design: A systematic literature review was conducted. From 71 reviewed, 23 studies were included (studies included questionnaire surveys and one interview, extracting barriers and facilitators, and analyzing using qualitative synthesis). Result: TFL indirectly and directly positively affects nurses’ work environment through mediators, including structural empowerment, organizational commitment, and job satisfaction. Nurses perceived that managers’ TFL behavior did not attain excellence in any of the included organizations, highlighting the necessity for additional leadership training to enhance the patient safety culture related to the non-reporting of errors and to mitigate the blame culture within the nursing environment. Conclusion: Bringing more focus to leadership education in nursing can make future nursing leaders more effective, which will cultivate efficient teamwork, a quality nursing work environment, and, ultimately, safe and efficient patient outcomes. This study was not registered.

## 1. Introduction

Patient harm caused by errors in healthcare is the leading origin of morbidity and mortality internationally [1]. Researchers are linking adverse patient safety outcomes to a lack of effective leadership, while relational leadership styles, like transformational leadership, continue to be associated with reduced adverse patient outcomes [2,3]. Nursing is dynamic and requires inspiring and engaging leaders and role models. However, the development of nurse leaders is challenging for the nursing profession.

Currently, nurses face a burnout epidemic rooted mainly in the work environment influenced by excessive workloads and a lack of organizational support and leadership [4]. Maben et al. (2022) reported that nurses globally face a heightened vulnerability to mental health issues and suicide, surpassing other occupational groups, while the COVID-19 pandemic has exacerbated the existing challenges in their work environment, further intensifying the already demanding conditions [5]. The engagement in emotional labor within the nursing profession exposes practitioners to a notable susceptibility to experiencing burnout, moral distress, and compassion fatigue. Prior to the onset of the pandemic, the international cadre of nurses was already confronting considerable hurdles, encompassing prolonged duty durations, rotation schedules, inadequate staffing, and periodically arduous situations [5,6,7]. Throughout the pandemic, nurses encountered a range of stress-inducing factors, including managing heightened public expectations and pressure, adapting to new work responsibilities, facing elevated mortality rates, dealing with the infectious nature of COVID-19, experiencing psychosocial stress, confronting the scarcity of personal protective equipment, handling demanding job requirements, and contending with inadequate psychological support [8]. At the same time, scholars have found poor working conditions for nurses and inadequate staffing to predict adverse patient outcomes based on the low-quality nursing job atmosphere and the absence of appropriate leadership styles [9,10].

Safety issues in care, such as adverse events, medication errors, falls, and surgery mistakes, have plagued healthcare systems internationally for decades. Several investigations have acknowledged healthcare environments as high-risk with a lack of safety culture, causing long-delayed discharge, disability, or even death [2,11]. Inherently, the nursing profession and current healthcare climate are chaotic, and a positive safety culture has been proven to come from a creditable and visible leader who supports patient safety behaviors [12]. It is important to recognize that nurses have the highest patient interaction, making nurse leaders central catalysts to positively influencing patient safety culture to reach safer patient outcomes [13,14].

The quality of the nursing work environment is an indicator of nurse satisfaction. A leader who involves staff fosters teamwork, rewards good performance, and encourages motivation can impact the quality of work life [15,16]. The leadership style describes how the leader interacts with others and can be categorized into two main styles: task-oriented and relational [17]. Historically, leadership theories started with the Great Man Theory during the Industrial Revolution with strong hierarchical leader-centric decision making, focusing on command-and-control, productivity, and seeing the organization as linear, operating like a machine [18]. This leadership style model in healthcare is no longer sustainable, as proven by a lack of change and persisting patient safety issues. Researchers have found that healthcare innovation requires nonlinear and emergent social processes that result in improved organizational outcomes [19]. In recent years, the two relational styles, transformational and transactional leadership, have been explored through nursing literature and have become high profile in general healthcare research.

Transformational leadership is composed of four key components. Firstly, “idealized influence” involves the leader behaving as a robust role model toward followers, demonstrating a work ethic and strong values while preaching the organization’s vision, thereby winning the staff’s trust and confidence [20]. The second type of behavior is referred to as “inspirational motivation”. It includes creating a compelling and inspiring vision for the future and communicating it to followers through emotionally charged speeches, vivid imagery, and captivating symbols. This encourages followers to strive to reach this shared vision, thus creating a deeper level of commitment and higher performance [17]. The third type of behavior is called “intellectual stimulation”. Intellectual stimulation encourages followers to think outside the box and consider different approaches to everyday issues, enabling them to devise innovative solutions to these problems [21]. The final category of behaviors is “individualized consideration”, including coaching, helping followers achieve goals, and providing a supportive climate. By carefully listening, leaders can help fulfill those needs [22]. For instance, some followers might require explicit guidance regarding how to get a job done, while others require the provision of needed resources so they can figure out the solution on their own. Nonetheless, TFL’s four behaviors construct a transformational leader if performed consistently and are found to bring respect and admiration by followers [23].

### 1.1. Rational

Healthcare systems are globally facing a crisis, with nurse shortage being a perennial issue. Nurses have the highest patient interaction, making nurse leaders central catalysts in positively influencing patient safety culture to reach safer patient outcomes [13]. At the same time, negative nursing work environments cultivate dissatisfied nurses who are likely to suffer from emotional exhaustion or burnout because of ineffective leadership [14]. Amidst these challenges, there is growing recognition of the potential impact of transformational leadership in healthcare settings.

Transformational leadership is characterized by its focus on relationship-building, empowering followers, and emphasizing shared values and vision. This leadership style has been found to positively affect various industries and sectors, including healthcare. However, there remains a gap in knowledge regarding its specific effectiveness in healthcare settings. A comprehensive analysis of the potential benefits of transformational leadership in the healthcare context is warranted. This systematic review aims to address this gap by investigating the effectiveness of transformational leadership and its potential to create better working environments, ultimately leading to improved patient outcomes. We have identified a crucial area of inquiry that has not been thoroughly examined in the existing literature—a systematic review that delves into the relationship between transformational leadership and its effects on both the working environment and patient outcomes. We have identified a single literature review from the preceding decade (2002–2012) that focused on the efficacy of transformational leadership in relation to both work environments and patient outcomes [24]. Considering this, our current investigation is oriented towards delving into scholarly works spanning the subsequent decade (2012–2022), with the intention of comprehensively examining the evolving discourse on this subject matter. By exploring and synthesizing the current body of knowledge on this topic, our study will contribute valuable insights to the field, allowing healthcare organizations to better understand the impact of transformational leadership and make informed decisions regarding their leadership practices.

The significance of this research lies in its potential to shed light on a promising approach to address the pressing challenges faced by healthcare systems—nurse shortage and dissatisfaction—through effective leadership strategies. By providing evidence-based insights, this review seeks to guide healthcare leaders in adopting transformational leadership practices to create a positive work environment for nurses, reducing emotional exhaustion and burnout, and ultimately enhancing patient care and safety.

In conclusion, the dearth of research on the relationship between transformational leadership, work environment, and patient outcomes in healthcare settings highlights the necessity of this review. By examining the effectiveness of transformational leadership and its potential impact on nurses’ well-being and patient outcomes, our study aims to fill this critical gap in knowledge and contribute to the advancement of healthcare leadership practices.

### 1.2. Objective and Research Question

Having delineated the rationale and imperative for conducting this systematic review, our primary aim was to search, retrieve, and critically evaluate all pertinent studies centered around the concept of transformational leadership, with a particular focus on its efficacy in fostering an improved working environment for nurses and influencing patient outcomes comprehensively and systematically.

Our aim was to synthesize and analyze studies, and therefore, we used the PICo framework for studies to determine a research question. PICo is the simplest of the frameworks to use for qualitative questions; it stands for Population, Interest, and Context and can be used to find a range of primary literature. The Population in our study is nurses; the Interest is transformational leadership, working environments, and patient outcomes; and the Context is hospitals. Based on the PICo framework, we formulated our research question as follows: “What is the impact of transformational leadership on staff nurse work environments and patient outcomes?”

## 2. Methodology

To effectively accomplish our aim and investigate our research question, we utilized a systematic review approach following the guidelines outlined in the PRISMA 2020 statement [25]. The PRISMA 2020 checklist is available in Appendix A. In the subsequent subsections, we provide a comprehensive overview of our methodology.

### 2.1. Eligibility Criteria

Each of the chosen studies incorporated in this systematic review had to fulfill specific inclusion criteria, as outlined in Table 1 provided below.

### 2.2. Information Sources and Search Strategy

We used the following databases to choose the articles: MEDLINE, CINAHL, and SCIENCE DIRECT. The search approach employed the Boolean operator OR between the keywords nurse, working environments, patients’ outcomes, and transformational leadership and comparable MeSH phrases. To refine the search, phrases with diverse meanings were joined using the Boolean operator AND. The search approach used on the EBSCO platform for the aforementioned databases is described in Table 2 We limited the search to journal articles in English with the full text available. However, numerous studies were rejected as they referred to other leadership styles than transformational leadership in addition to other healthcare settings than a nursing work environment.

### 2.3. Selection of Studies Process

Two researchers (the first two authors) conducted independent searches, retrievals, and selections of studies, initially based on three primary criteria: (a) the presence of primary research, (b) the inclusion of transformational leadership as a topic, and (c) relevance to nursing care. Subsequently, additional criteria, such as peer-reviewed articles published in journals or conference proceedings, as well as the publication date, were employed for further refinement. Upon completing the initial selection process, the two researchers engaged in discussions and compiled a list of prospective articles. This list was shared with four other researchers, who collectively determined the final articles to be included in the review, making any necessary additions or removals as deemed appropriate.

### 2.4. Data Collection Process

The data from the selected studies were independently collected by two researchers. They extracted the components, items, statements, or competencies that had achieved consensus among experts during the final round of each study. Specifically, the following data from each study were extracted: title of the study, authors’ names, publication year, study design, tools, sample characteristics, and summary of main findings and results. Subsequently, the researchers thoroughly reviewed the extracted data multiple times and proceeded to code and identify overarching themes.

### 2.5. Synthesis Methods

The data were synthesized by content analysis, and the findings were categorized into themes. After carefully examining the results and findings section of a chosen article, an initial set of codes was created. These codes underwent further improvement as more articles were analyzed. Each line of text was assigned a code, and a code tree was utilized to identify emerging themes. From the interpreted meanings, sub-themes were derived and combined. These sub-themes underwent further analysis and were eventually condensed into a single overarching theme. Content analysis can aid in the identification and summarization of submerging key elements within a large body of data during the review process [26]. The themes of the effectiveness of TFL in the nursing environment were organized according to the content analysis suggested by Zhang and Wildemuth (2009) [27].

To ensure the validity of the results, a two-level quality assurance process was implemented. The authors of this paper independently followed the review procedure, including coding, categorization, revisiting the studies, and refining the codes and categories. Subsequently, they convened, engaged in discussions, refined the analysis, and finalized the results.

## 3. Results

**Figure 1:**
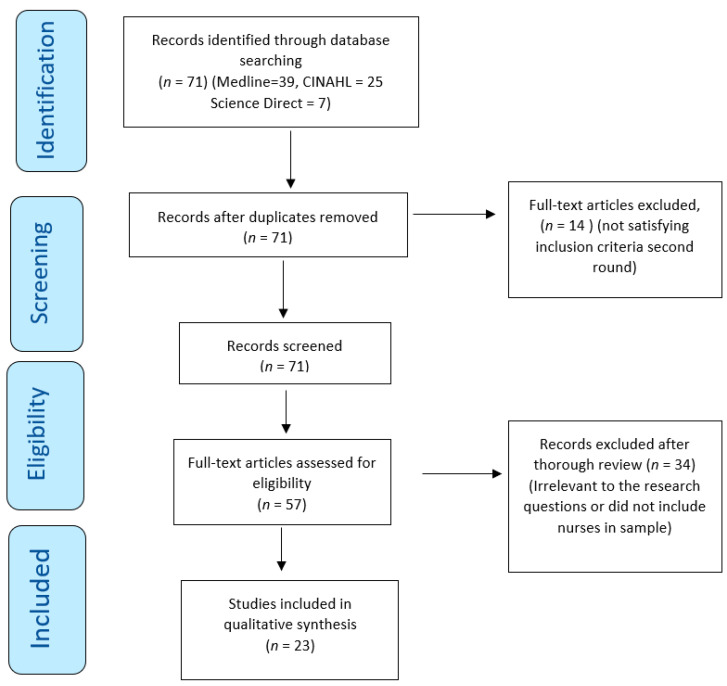
PRISMA flowchart with the search strategy of the systematic review.

This review was conducted in accordance with the PRISMA statement (Figure 1) [25], which provides a set of guidelines for conducting reviews and meta-analyses in a comprehensive and systematic manner.

### 3.1. Studies Selection

The initial search process resulted in 71 articles related to transformational leadership. There were no duplications (Figure 1), and therefore, 71 articles were included for advance screening. Fourteen (14) articles did not relate to nurses’ work environment and were omitted. Two researchers thoroughly reviewed the remaining 57 articles independently. From this process, 34 articles were excluded as they did not satisfy the criteria for inclusion. The final number of articles that met the criteria for inclusion was twenty-three (23).

### 3.2. Studies Characteristics

These 23 articles were conducted in various countries and assessed the effect of transformational leadership in a nursing clinical work environment. Most of the studies included a multifactor leadership questionnaire to evaluate nurses’ perceived effectiveness of transformational leadership (1–10, 13, 14, 16, 18, 19, 22, 23). Further information about the articles, such as author, year, tool, methodology, sample, and main results, is described in Table 3 below.

### 3.3. Study Assessment

The quality of the articles included in this review was checked by the Joanna Briggs Institute Qualitative Assessment and Review Instrument Critical Appraisal Checklist. The Joanna Briggs checklist evaluates the methodological quality of a study while determining the possibility of an indication of bias in its conduct, design, and analysis. As can be seen from Table 3, there were 21 cross-sectional studies (1–11, 13–19, 21–23), 1 descriptive–correlational study (12), and 1 qualitative study (20).

All the included studies largely adhered to the Joanna Briggs criteria, providing comprehensive and detailed descriptions of their respective methodologies and procedures Table 4, Table 5 and Table 6. However, it was observed that two of the cross-sectional studies did not explicitly outline any specific strategies to address the stated confounding factors. Nevertheless, as Dekkers et al. (2019) argue, confounding is not dichotomous but rather a continuum where varying degrees of confounding influence can exist [28]. Furthermore, in accordance with the Joanna Briggs guidelines, the qualitative study failed to disclose the researcher’s cultural or theoretical standpoint, as well as the potential influence of the researcher on the research process. It is worth noting that such omissions are common in qualitative studies, where the focus is on understanding the subjectivity of the participants and allowing their perspectives to emerge naturally.

### 3.4. Results of Synthesis

Two major themes emerged, effectively addressing the research questions. Within each theme, several categories were identified, shedding light on the multifaceted nature of the topic under investigation. The themes and their corresponding categories were as follows.

Theme 1: Staff nurses’ work environment:

1. Job Satisfaction and Organizational Commitment;
2. Reduce Nurse Retention;
3. Nurses’ Empowerment and Autonomy;
4. Nurses’ Compliance with Safety Measures.

Theme 2: Patients’ outcomes:

1. Patient Safety Culture;
2. Reporting Adverse Events.

#### 3.4.1. Job Satisfaction and Organizational Commitment

Various studies that investigated the mechanism of TFL detected its strong influence on employee attitudes and behaviors in nursing. Nurses’ work attitudes are reflected in their levels of job satisfaction and organizational commitment [29,30]. It was clear from the literature that TFL frequently positively influenced nurses’ work environment by indirectly increasing job satisfaction [31,32,33,34]. Employees’ positive perception of jobs and organization is revealed through job satisfaction [30]. Researchers link TFL and empowerment to the establishment of self-determination and competency, which is proven to impact job satisfaction, suggesting the direct relationship between nurse empowerment and nurse job satisfaction, enhancing the quality of the nurses’ work environment [9,32].

There is also evidence to construct a strong link between organizational commitment and job satisfaction. Interestingly, the statistics showed that nursing staff committed to their organization with a strong sense of loyalty and dependence also had higher levels of job satisfaction [29,33]. Further, higher levels of organizational commitment and job satisfaction were also associated with increased health status in the nurses [33]. More specifically, TFL was related to more excellent supervisor support, increasing job satisfaction among the nurses, and resulting in more significant organizational commitment [29]. In a study examining the effectiveness of TFL in the environment of elderly care, TFL was found to effectively strengthen the nursing staff’s sense of belonging to the organization, reducing their burnout. The clan culture established through TFL effectively influenced organizational commitment and job satisfaction, where the atmosphere of a home culture created within their work environment promoted the intrinsic values of nursing staff while improving cohesion between the nurses and the quality of care [33]. However, TFL was found to have a direct positive effect on organizational commitment [33,35].

#### 3.4.2. Reducing Intention to Leave the Job/Organization

Studies also found that TFL can reduce the nurses’ intent to leave the job, which is closely related to the previous category, as job dissatisfaction can be the primary precursor of nurses’ intent to leave [29]. The literature generally highlights that the TFL style shapes employees’ perceptions and feelings around their nursing managers and affects their desire and obligation to maintain the intent to stay in their organization [36]. A recent cross-sectional study examining 645 nurses working during the COVID-19 pandemic found that a supportive workplace culture can construct an adaptive mechanism through which transformational leaders can improve retention [37]. Additionally, the literature found TFL to decrease emotional exhaustion amongst nurses by encouraging a spiritual climate, indicating that a positive spiritual climate facilitated through TFL can reduce burnout and decrease nursing staff’s intent to leave [31]. However, there was insufficient evidence proving a direct correlation between TFL and staff nurses’ decision to stay or leave their job [33,35], but it was suggested that TFL has the potential (but not the primary factor) to slow down attrition and retain nurses by improving job satisfaction and organizational commitment, creating a positive work environment and increasing nurses’ probability of staying [35]. TFL seems to not act directly on job satisfaction or intent to stay but rather create a multifaceted positive work environment leading to a quality nursing environment. Consequently, it was reported that TFL indirectly influenced willingness to stay by positively influencing staff organizational commitment and job satisfaction [29,33,35].

#### 3.4.3. Nurses’ Empowerment and Autonomy

Literature highlights that the TFL style within nursing can give staff nurses increased autonomy through empowerment strategies and meaningful participation in decision-making [30,31,36]. In turn, TFL-facilitated empowerment has been proven to increase employee commitment within their units by delegating power to nurses, leading to increased authority within their work environment [30,36]. Empowerment through decision-making involvement via removing formal organizational barriers has been found to reduce powerlessness in the nurse work environment, reducing job burnout and increasing job satisfaction [30]. RN-MD collaboration and teamwork within and across units were thought to be necessary for the nurse’s autonomy [38]. Further, the literature relates to the concept that a well-functioning patient safety climate requires nurses with autonomy to deal with problems regarding patient safety while proposing specific solutions and getting support and encouragement from organizations to facilitate patient safety-based innovations [39].

TFL and transactional leadership behaviors were found to affect empowerment amongst the nursing staff positively. However, TFL behaviors allowed nursing managers to reach even higher levels of success without congruence and reward, embedding empowerment into the clinical environment [40]. Some studies also identified the empowerment subscale, autonomy, as the statistically significant predictor of commitment, indicating that managers can engage nurses in appropriate decision making about patient care and safety in their work environment [30,36]. Management that does not accept decision-making participation dissembles empowerment, which frustrates and makes staff dependent on an authoritarian structure [36].

#### 3.4.4. Nurses’ Compliance with Safety Measures

Lievens and Vlerick (2014) found a strong association between TFL and nurse safety compliance [41]. The more transformational the leader was perceived, the more the nursing staff participated and complied with patient safety practices. Further, staff nurses’ structural empowerment also experienced a significant correlation with the degree to which they perceived nursing managers’ (NMs) TFL behaviors [36,40]. Research also suggested that when nurses perceived their TFL to facilitate an innovative work climate, they automatically contributed to developing innovation behaviors [39]. Previously mentioned research suggested that nurses need to feel a part of their work environment. However, countries where staff are hesitant to challenge authority create a reluctance to change, and compliance can breed a lack of stimulation [31]. It was reported that nurse managers should be trained to challenge nurses to resolve problems and specialize their competence to foster innovation and grow talents and creativity [36].

Lievens and Vlerick (2014), in their cross-sectional study which included 145 nurses, also found intellectual stimulation to strongly impact knowledge-related characteristics, suggesting an indirect link between safety performance and TFL through skills and ability demands, where the more knowledge-related job characteristics were perceived, the more nurses complied with safety rules [41]. Skill utilization or intellectual stimulation was further found to be the strongest single predictor of work engagement, compared to TFL, where nurses appreciated opportunities for personal development, learning new things, and achieving something meaningful, encouraging work engagement [2,42].

Patients’ outcomes:

The literature shows a positive relationship between TFL and the improvement of patient safety climate and culture, emphasizing that nursing managers are key to developing a safety climate and maintaining a culture of patient safety, preventing adverse events.

#### 3.4.5. Increase Patient Safety Culture

There was a significant prevalence of findings reporting TFL to facilitate patient safety either directly [2,9,38,42] or indirectly [32,39,41]. Seljemo et al. (2020), in their cross-sectional study, questioned 156 nurses; Ree and Wiig (2019), also in a cross-sectional design study, questioned 139 nurses and found TFL to be the strongest predictor of patient safety culture and overall perception of patient safety compared to job demands and resources [2,42]. This was suggested to result from TFL having a positive direct effect on the psychosocial work environment. Further evidence also links TFL directly to quality patient outcomes, reducing the possibility of adverse patient outcomes and increasing the quality of care [9].

Patient safety culture includes themes such as teamwork within units, managers’ support, organizational learning, overall perceptions of safety, feedback and communication openness about the error, frequency of events reported, staffing, handoffs and transitions, and non-punitive response to errors. “Teamwork within units” generally had a common positive perception amongst the nurses, indicating collaboration within their units as effective within TFL [38,43,44]. Anselmann and Mulder (2020) asked 183 geriatric nurses in their cross-sectional study, and they support the above, finding that TFL has a positive impact on team performance when a safe climate is fostered [45]. Even though nurses found cohesion within their units, literature revealed a common theme of insufficient “teamwork between units”, indicating that each unit had an independent culture [38,43,44]. Further, a generally weak perception of the effectiveness of RN-MD collaboration was also observed [38,43].

Researchers stressed the necessity of having efficient teamwork between units and on a multi-professional level to create an effective patient safety culture [9]. Another reoccurring subdimension, “feedback and rewarding”, was also identified as a weak component of TFL in relation to patient safety culture, illustrating a lack of adaptation and implementation of TLF behavior [9,43,46]. The TFL nursing manager generally seemed to conduct insufficient work around feedback and rewards, resulting in staff nurses not being encouraged and ensuring that medical errors were prevented and learned from [43,46].

#### 3.4.6. Reporting Adverse Events

Adverse events can result in patient disability or death, prolong the time necessary to provide care, and increase healthcare costs and patient dissatisfaction [47]. However, a part of the literature showed that when TFL and transactional leadership were compared, reporting errors without blame and discussing errors openly were the two initiatives that transactional leadership implemented better than TFL [40,48]. A significant finding in the literature was the reoccurring theme of weak patient safety culture in relation to “non-punctual reporting of adverse events” in hospitals with TFL, where staff nurses rarely reported occurring medical errors to their NMs [34,44,46,48,49]. In a Finnish study, one in four nurses showed to not have reported one or more medication errors using their units’ adverse event registration system [46]. Tekingündüz et al. (2021), in a cross-sectional study with 150 participating nurses, also found a significant weakness in their organization’s patient safety culture, where 52.7% of the nurses did not report any adverse events in the last 12 months, 31.3% reported 1–2 adverse events while 10% reported 3–5 adverse events [49]. Further, in a qualitative study, the eleven nurse manager participants expressed the importance of nursing staff reporting the occurrence of adverse events to detect why each event happened and identify patient safety risks and solutions [50]. There was evidence to suggest that nurses reported that the occurrence of errors only sometimes led to a positive change, whereas at other times, it did not lead to any change, and errors were repeated [38]. The literature explained blame culture and fear in the nurse’s work environment as a factor distancing them from punctuative reporting of medical errors [46,49,50]. It was suggested by researchers that nursing staff were not encouraged to report and discuss adverse events openly and blame-free [48,49,50]. This involves handling adverse reports by nursing managers without making nursing staff feel guilty.

Managers reported that a culture where it is recognized that everyone makes mistakes is imperial, while it was observed that nurses tended to report other colleagues’ mistakes compared to their own [50]. Further, nursing managers noticed that nursing staff may blame themselves for a patient safety incident where they feel ashamed and worry about their colleague’s perception of them [49]. These perceptions were confirmed by nursing staff in another study, expressing their tendency to avoid reporting due to fear of punishment, humiliation, damage to reputation, disciplinary action by a licensing board, malpractice lawsuits, and limited follow-up after reporting loss of job [48]. Tekingündüz et al. (2021) also found the defect in reporting medical errors to be rooted in nurse’s fear of punishment and lack of confidentiality [49]. Generally, fear was perceived as a major reason for not reporting adverse events, and nursing managers saw this as a barrier to the effectiveness of their leadership and the attempt to develop their operational models to improve patient safety [46,49,50]. However, visionary leadership styles such as TFL correlate positively with both incident reporting and patient safety outcomes. Additionally, TFL is linked to improved patient safety, including reduced mortality rates, fewer medication errors, lower incidences of pneumonia and urinary tract infections, and fewer patient falls, attributed to the leaders’ approach of using errors as chances to enhance processes and promoting the reporting of near misses and adverse events [17,51].

Interestingly, a part of the literature showed that when TFL and transactional leadership were compared, reporting errors without blame and discussing errors openly were the two initiatives that transactional leadership implemented better than TFL [40,48]. These findings confirm the weakness around reporting adverse events and blame culture within TFL units.

## 4. Discussion

This review has collectively reviewed literature that has examined the effectiveness of transformational leadership (TFL) in a nursing work environment and patients’ outcomes. TFL has a complex, interconnected effect on nurses’ intrinsic environment and patient outcomes.

Nurses’ Work Environment:

The literature revealed substantial evidence that TFL can significantly enhance nurses’ psychosocial work environment by indirectly increasing job satisfaction. Three significant mediators between TFL and job satisfaction were nurse empowerment, organizational commitment, and spiritual climate, which altogether were thought to prevent retention in nursing [29,30,31,33,34,35,37]. Simultaneously, TFL was not the primary factor in job satisfaction but instead a facilitator and constructor of structural empowerment, organizational commitment, and spiritual climate. It is, therefore, evident that the literature revealed a positive domino effect that transformational leaders in nursing can generate. Generally, the literature revealed a strongly positive relationship between TFL and workplace culture in nursing [33,37]. Specific TFL attributes created an inclusive and supportive work environment, either directly or indirectly enhancing the nurses’ work environment and decreasing the risk of nurse burnout [37,52]. Nurses continuously reported managers’ support as a particularly important resource in their work environment, where establishing a high-quality relationship with their leaders was seen as imperial for patient safety culture [38,42].

The correlation observed between supportive leadership and favorable patient safety outcomes underscores the significance of Transformational Leaders (TFLs) possessing a comprehensive grasp of patient safety protocols, as well as recognizing the pivotal role played by bedside nurses in advancing improved safety outcomes. [17]. More specifically, managers’ support was also found to reinforce innovative behavior [39], increase job satisfaction [35,37], and even be the primary factor in a positive work environment, compared to TFL [29]. Conversely, the literature also described managers’ support as a core transformational behavior, where the more transformational the leader was perceived, the more the staff nurses experienced individual support in their clinical environment [29,42,46]. As concluded by the literature, TFL is not the primary factor but rather a mediator to job satisfaction, which was determined as an essential nursing outcome, shadowing quality work environment and may be an effective retention strategy in nursing. Previous studies confirm that safety outcomes are improved when workplace empowerment takes place in a positive nurse–leader relationship based on trust and respect, where they, together, work toward a patient safety culture [53].

Therefore, incorporating transformational leadership in nursing has numerous implications, with a direct and positive impact on job satisfaction. By nurturing a sense of purpose, providing support and empowerment, and promoting individual growth, transformational leaders create a fulfilling work environment that motivates nurses to excel. As nurses experience greater job satisfaction, patient care quality also improves, resulting in cooperative success for healthcare organizations, nursing staff, and the patients they serve.

Patients’ Outcomes:

The connection between supportive leadership and positive patient safety outcomes points to the importance of the TFL’s understanding of patient safety processes and the role of bedside nurses in promoting better safety outcomes [38]. However, several researchers reported not having a visible leader [43], which is documented as essential for patient safety changes to occur [53].

Researchers are linking negative patient safety outcomes to a lack of effective leadership, while relational leadership styles like transformational leadership continue to be associated with reduced adverse patient outcomes [17]. However, TFL nursing managers were repeatedly reported by the staff nurses only to communicate errors and problems after the adverse event, waiting for the event before resolving problems and taking proactive action [36,50]. Literature highlights that organizations that have successfully created a non-blame culture have better patient safety outcomes because the staff are encouraged to report errors, unsafe practices, and adverse events, perceiving safety around seeking help and assistance without threat [54]. Therefore, avoiding a blame culture and developing a reporting system serves as a proactive approach to identifying and mitigating risks, ultimately preventing errors and recurring mistakes, which, when left unaddressed, can result in significant social and economic burdens due to fatalities and preventable incidents [51] Additionally, developing a safety culture through managers’ interdisciplinary walkabout safety rounds has been associated with safety outcomes [17].

Transformational leadership in nursing has far-reaching implications for patient outcomes and care quality. By fostering a collaborative and patient-centered approach, empowering nursing staff, encouraging continuous learning, and promoting a culture of excellence, transformational leaders enhance the overall care experience for patients. Ultimately, the positive impact of transformational leadership on patient outcomes establishes it as a key factor in ensuring the delivery of high-quality healthcare services in nursing settings.

This literature review enriches nursing practice and research in a time where nursing leaders are sought to have an important and prominent role in healthcare policy development and improvement. Increased demand and complexity of patient care require effective and competent leadership skills and an understanding of TFL’s function in the current healthcare environment. Even though literature has constructed the idea of the nexus between patient safety and leadership, patient safety outcomes are unlikely to improve without facilitating and fostering the professional growth of future leaders. Additionally, factors influencing organizational job satisfaction and organizational commitment are significantly under the influence of TF nurse leaders. Therefore, healthcare organizations and the educational sector should invest in leadership training and curriculum to implement it further into nursing to support and ensure safe, quality work environments for both nurses and patients.

## 5. Limitations of the Study

This literature review predominantly incorporated quantitative research methodologies, which, in certain instances, can present challenges in contextualizing a phenomenon comprehensively, as the data may not always possess the robustness required to elucidate intricate issues. Additionally, it should be noted that the review’s scope was confined to studies published exclusively in the English language, with no inclusion of relevant content from the grey literature beyond the stipulated publication sources, and unpublished dissertations were also omitted from consideration. Consequently, it is essential to acknowledge that this review may not provide a fully representative overview of all pertinent scholarship within the field.

## 6. Conclusions

Despite the global recognition and attempted implementation of TFL in healthcare, the statistics still show that TFL is yet to be mastered within nursing. The strong relationship between TFL, structural empowerment, job satisfaction, and organizational commitment signify that an improved quality work environment may be the most essential element to enhance job effectiveness and patient safety in nursing. TFL is a vital facilitator that could help healthcare to improve job satisfaction and reduce adverse events. Evidence suggests that nursing managers who possess effective TFL attributes are likely to influence their nursing staff’s satisfaction and mitigate the risk of burnout by establishing a supportive and inclusive work environment directly or indirectly. Focusing on the adoption of a blame-free culture through effective leadership is likely to break down barriers to safety culture, which has resulted in poor patient care worldwide. Patient safety outcomes rely on a well-established patient safety culture, which is most influenced by the bedside nurse, either directly or indirectly. With effective leadership engagement and education, emerging nursing leaders can be supported while the nursing team can be empowered to make the necessary changes to reach levels of excellence within their units. It is important to comprehend that leaders are not just in executive and senior positions but include any part of the healthcare team that is influential to patient care. Effective TFL engagement has the potential to enhance patient safety, where it is conveyed that all healthcare workers, from executive to bedside nurses, participate in a positive safety culture.

## Figures and Tables

**Table 1:** Inclusion/Exclusion Criteria.

Inclusion Criteria	Exclusion Criteria
Peer Reviewed	The sample does not include nurses
Primary sources	Secondary sources
Include nurses in the study sample	Not written in the English Language
Written in English	Published earlier than 2012
Published between 2012 and 2022 (to capture a broad range of research on our topic within the last decade)	

**Table 2:** Search approach.

Population		Interest		Context
(TL (“Registered Nurse” OR “RN” OR “Nurs * p *” OR “Nursing staff” OR “Clinical nurse” OR “Nurse specialist” OR “Nurse clinician” OR “Nursing care provider” OR “Nursing team member”) OR AB (“Registered Nurse” OR “RN” OR “Nurs * p *” OR “Nursing staff” OR “Clinical nurse” OR “Nurse specialist” OR “Nurse clinician” OR “Nursing care provider” OR “Nursing team member”) OR DE “Nursing”)	AND	(TL (“Transformational leadership” OR “TFL” OR “Transformational leader*” OR “Transformational manager*”) OR AB (“Transformational leadership” OR “TFL” OR “Transformational leader *” OR “Transformational manager *”) OR DE “Transformational leadership”)	AND	(TL (“Work Environment” OR “Working Conditions” OR “Workplace” OR “Job Satisfaction” OR “Patient Outcome” OR “Health Outcome” OR “Treatment Outcome”) OR AB (“Work Environment” OR “Working Conditions” OR “Workplace” OR “Job Satisfaction” OR “Patient Outcome” OR “Health Outcome” OR “Treatment Outcome”) OR MM (“Working Environments” OR “Outcome Assessment, Health Care”))

* The asterisk in Ebsco platform wildcard in search finds words with a common root.

**Table 3:** Articles Description.

Authors and Year	Tool	Methodology	Sample	Main Results
Boamah, S., Spence Laschinger, H., Wong, C., and Clarke, S., 2018.	TFL—Multifactor Leadership Questionnaire (MLQ) Job satisfaction—Global Job Satisfaction Scale (GJSS) Conditions Of Work-Effectiveness II (CWEQ-II) Nurse-assessed adverse patient outcomes	Cross-sectional	378 nurses	Significant indirect relationship between TFL and adverse patient outcomes. The level of staff empowerment strongly influences the job satisfaction of nurses. Nurses perceived TFL behaviors of managers to be moderate.
2. Asif, M., Jameel, A., Hussain, A., Hwang, J., and Sahito, N., 2019.	TFL (7-item scale) Structural empowerment (12-item scale) Job satisfaction (3-item scale) Adverse patient outcomes (5-item scale)	Cross-sectional	386 nurses	TFL behavior was found to have a positive effect on patient outcomes, decreasing the likelihood of unfavorable outcomes and improving the overall quality of care. The mediator between TFL and these desired patient outcomes was structural empowerment and job satisfaction. Nurses perceived TFL behaviors of managers to be high.
3. Lappalainen, M., Härkänen, M., and Kvist, T., 2020.	TFL—Transformational Leadership Scale (TLS) Medication error—Medication Safety Scale (MSS)	Cross-sectional	161 nurses	Nurses did not perceive managers to fully adapt TFL behaviors. Support for professional development was strongly perceived. Giving feedback and rewarding was the weakest area of TFL. TFL related the strongest to medication safety through the management of the nursing process.
4. Seljemo, C., Viksveen, P., and Ree, E., 2020.	TFL—The Global Transformational Leadership Scale (GTL) Job demands and resources—Short Inventory to Monitor Psychological Hazards (SIMPH)/Job Demands–Resources model Patient safety culture—Nursing Home Survey On Patient Safety Culture (NHSOPSC)	Cross-sectional	156 nurses	The speed of work and the amount of emotional strain on employees had a negative effect on patient safety culture. The impact of TFL on patient safety culture and overall perception of patient safety was the most significant factor.
5. Ree, E. and Wiig, S., 2019.	TFL—The Global Transformational Leadership Scale (GTL) Job demands and resources—Short Inventory to Monitor Psychological Hazards (SIMPH)/Job Demands–Resources model Patient safety culture—Nursing Home Survey On Patient Safety Culture (NHSOPSC)	Cross-sectional	139 nurses	TFL was responsible for 35.7% of variance in patient safety culture TFL and job resources positively related to work engagement Skill utilization was the strongest single predictor of work engagement compared to TFL.
6. Lievens and Vlerick, P., 2014.	TFL—Multifactor Leadership Questionnaire (MLQ) Knowledge-related job characteristics—Work Design Questionnaire (WDQ) Safety performance and compliance	Cross-sectional	152 nurses	The more transformational the leader was perceived, the more nursing staff participated and complied with patient safety. Indirect link between TFL and safety performance via knowledge-related job characteristics. TFL can influence perceptions of knowledge-related job characteristics of followers through intellectual stimulation.
7. Asiri, S., Rohrer, W., Al-Surimi, K., Da’ar, O., and Ahmed, A., 2016.	TFL—MLQ Psychological empowerment Employee commitment	Cross-sectional	332 acute care nurses	Highest perceived leadership style was TFL, with inspirational motivation and idealized attributes being high. Transactional leadership and laissez-faire leadership had a more positive and significant effect on commitment than TFL. Having a TFL style of management can increase employee devotion through granting authority, as well as involving staff in the decision-making process.
8. Y Tekingündüz, S., Yıldız, E., and İnci, R., 2021.	TFL—Global Transformational Leadership Scale (GTFLS) Organizational trust—organizational trust scale Organizational identification—Organizational identification Job stress—Job Stress Scale (JSS) 7 items	Cross-sectional	150 nurses	Non-punctuative reporting medical errors: 52.7% no adverse events reported in 12 months, 31.3% reported 1–2 adverse events and 10% reported 3–5 adverse events. Positive relationship between organizational identification, organizational trust, and TFL.
9. Khan, B., Quinn Griffin, M., and Fitzpatrick, J., 2018.	TFL—MLQ-5X Structural empowerment—C WEQ11	Cross-sectional	181 clinical nurses	Statistically significant correlation between staff nurses’ perception of managers’ TFL behaviors and their structural empowerment as frontline staff. A negative correlation was found between structural empowerment and staff nurses’ perception of NMs’ laissez-faire leadership.
10. Weng, R., Huang, C., Chen, L., and Chang, L., 2015.	TFL—adopted from earlier studies 19 items Patient safety climate	Cross-sectional	439 nurses	Manager support was highly associated with nurse innovation behaviors. TFL had a significantly positive effect on nurse innovation behavior. TFL was strongly related to both innovation climate and patient safety climate.
11. El-Demerdash, A., 2018.	Patient safety culture—AHRQ (2004) TFL—Forces of Magnetism questionnaire	Cross-sectional	324 nursing staff	TFL was found to have a high magnetic force. Strong positive correlation between TFL and patient safety culture. Management support for patient safety was reported as highly important.
12. Kvist, T., Mäntynen, R., Turunen, H., Partanen, P., Miettinen, M., Wolf, G., and Vehviläinen-Julkunen, K., 2013.	Patient safety culture—HSPSCPatient satisfaction—RHCS	Descriptive correlational	2566 patients 5778 nursing staff and leaders	Highest score of managers TFL behavior: support for professional development. Feedback and rewards were the weakest for nurse managers. Awareness of the work of nursing directors was low. Patient satisfaction outcome was the only factor exceeding target level.
13. Choi, S., Goh, C., Adam, M., and Tan, O., 2016.	TFL—MLQ Job satisfaction Empowerment	Cross-sectional	200 clinical nurses	TFL showed a significant indirect positive effect on job satisfaction. TFL was directly related to fostering structural empowerment, which in turn affected job satisfaction positively.
14. Brewer, C., Kovner, C., Djukic, M., Fatehi, F., Greene, W., Chacko, T., and Yang, Y., 2016.	Organizational commitment Job satisfaction TFL	Cross-sectional	1037 newly licensed registered nurses	TFL did not have direct impact on intent to stay. Organizational commitment, job satisfaction, RN-MD collaboration, and mentor support had a positive effect on the intent to stay. TFL had non-significant direct probability of increasing organizational commitment. TFL was not found to be a significant predictor of job satisfaction.
15. Wu, X. et al., 2020.	Spiritual Climate Scale Emotional Exhaustion ScaleIntent to leave—Turnover Intention Scale	Cross-sectional	319 nurse clinicians	Nurse staff experienced moderate levels of TFL. Nurses frequently felt emotional exhaustion, burnt out, and had thoughts of leaving profession. Strong relationship between TFL and spiritual climate, where spiritual climate had a mediating effect on TFL’s ability to reduce burnout and intention to leave.
16. Xie, Y. et al., 2020.	TFL—research questionnaire Clan culture Organizational Culture Measurement Scale Organizational commitmentJob satisfaction	Cross-sectional	217 geriatric nurses	TFL and clan culture together explained job satisfaction amongst nursing staff. Organizational commitment, job satisfaction, and professional identity had a significantly positive effect on willingness to stay.
17. Boamah, S.A., 2022.	MLQ-5X—shorter rate form Workplace culture six-item measure created for the study Job satisfaction—GJS Burnout—MBI- COVID-19—measured with six items around demand and pressure	Cross-sectional	645 nurses	TFL had a strong, significantly positive effect on job satisfaction and workplace culture and a negative effect on burnout. TFL was found to, directly and indirectly, improve work environment. Direct, robust positive relationship between TFL and workplace culture. TFL can influence staff nurses’ satisfaction and mitigate the risk of burnout by establishing a supportive and inclusive work environment.
18. Anselmann, V. and Mulder, R.H., 2020.	TFL—GTL Team performance Team climate Knowledge sharing	Cross-sectional	183 geriatric nurses	TFL facilitated a safe team climate, which allowed knowledge sharing and reflection on processes and tasks. This was found to increase the team performance, including effectiveness and innovativeness. TFL enhanced learning activities of teams, which in turn affects their performance and outcomes positively.
19. Yilmaz, A. And Duygulu, S., 2020.	Leadership Practices Inventory (LPI) Patient safety culture—HSOPSC	Cross-sectional	Nursing managers and nursing staff	Nursing manager’s perception of their own TFL was higher than staff nurses. Lowest sub-dimension was the sub-dimensions of staffing, non-punctuative response to errors, and frequency of errors reported by both parties, at lower than 50%, indicating PSC weakness.
20. Liukka, M., Hupli, M. and Turunen, H., 2017.	Semi-structured interview	Qualitative study	11 nurse managers	Adverse events reporting reform leaving dysfunctional operational models. Encouraging nursing staff’s openness around adverse events by establishing a blame-free culture. Blame and shame—a challenge to recognize adverse events.
21. Wagner, A. et al., 2019.	TFL—MLQ Patient safety—PSQ	Cross-sectional	1355 nurses and pharmacists	Non-significant effect on error reporting compared to transactional leaders who showed higher levels of good reporting practices. Even though TFL was main behavior, no preventative actions were mentioned in incident reports.
22. Lin et al., 2015.	Multifactor Leadership Questionnaire Karasek’s Job Content Questionnaire (JCQ) Occupational Stress Indicator (OSI) Organisational Commitment Questionnaire (OCQ) General Health Questionnaire	Cross-sectional	651 nurses	Based on the main hypotheses of the research, the results revealed a positive relationship between nursing transformational leadership and general health status. The supervisor support plays a mediating role between transformational leadership styles and job satisfaction. Supervisor support has a dramatic influence on employees’ job satisfaction compared with other factors.
23. ALFadhalah and Elamir, 2021.	Multifactor Leadership Questionnaire Organizational Description Questionnaire Annual quality indicators from the hospitals	Cross-sectional	1626 health care workers	In each hospital, 66.4% to 87.1% of participants identified their hospital’s organizational culture as transformational, whereas 41 out of 48 departments were identified as having a transformational culture. The differences between leadership style and organizational culture were statistically significant for four of the hospitals. For most of the quality indicators, there was a positive but non-significant, correlation with leadership style.

**Table 4:** JBI Critical Appraisal Checklist for Analytical Cross-Sectional Studies.

Authors and Year	Q1	Q2	Q3	Q4	Q5	Q6	Q7	Q8
Boamah, S., Spence Laschinger, H., Wong, C., and Clarke, S. (2018)	√	√	√	√	√	√	√	√
Asif, M., Jameel, A., Hussain, A., Hwang, J., and Sahito, N. (2019)	√	√	√	√	√	√	√	√
Lappalainen, M., Härkänen, M., and Kvist, T. (2020)	√	√	√	√	√	√		√
Seljemo, C., Viksveen, P., and Ree, E. (2020)	√	√	√	√	√	√	√	√
Ree, E. and Wiig, S. (2019)	√	√	√	√	√	√	√	√
Lievens and Vlerick, P. (2014)	√	√	√	√	√	√	√	√
Asiri, S., Rohrer, W., Al-Surimi, K., Da’ar, O., and Ahmed, A. (2016)	√	√	√	√	√	√	√	√
Y Tekingündüz, S., Yıldız, E., and İnci, R. (2021)	√	√	√	√	√	√	√	√
Choi, S., Goh, C., Adam, M., and Tan, O. (2016)	√	√	√	√	√	√	√	√
Khan, B., Quinn Griffin, M., and Fitzpatrick, J. (2018)	√	√	√	√	√	√	√	√
Weng, R., Huang, C., Chen, L., and Chang, L. (2015)	√	√	√	√	√	√	√	√
El-Demerdash, A. M. S., Elhosany, W. A., and Hefny, M. A. M (2018)	√	√	√	√	√		√	√
Brewer, C., Kovner, C., Djukic, M., Fatehi, F., Greene, W., Chacko, T., and Yang, Y. (2016)	√	√	√	√	√	√	√	√
Xie, Y. et al. (2020)	√	√	√	√	√	√	√	√
Boamah, S.A. (2022)	√	√	√	√	√	√	√	√
Anselmann, V. and Mulder, R.H. (2020)	√	√	√	√	√	√	√	√
Yilmaz, A. and Duygulu, S. (2020)	√	√	√	√	√	√		√
Wagner, A. et al. (2019)	√	√	√	√	√	√	√	√
ALFadhalah, T. and Elamir, H. (2021)	√	√	√	√	√		√	√
Liukka, M., Hupli, M., and Turunen, H. (2017)	√	√	√	√	√	√	√	√
Lin, PY., MacLennan, S., and Hunt, N (2015)	√	√	√	√	√	√	√	√

**Table 5:** Risk of Bias Assessed by the Joanna Briggs Institute Critical Appraisal Checklist for Qualitative Study Results.

Authors and Year	Q1	Q2	Q3	Q4	Q5	Q6	Q7	Q8	Q9	Q10
Liukka, M., Hupli, M., and Turunen, H. (2017)	√	√	√	√	√	No	No	√	√	√

**Table 6:** JBI Critical Appraisal Checklist for Studies Reporting Prevalence Data Results.

Authors and Year	Q1	Q2	Q3	Q4	Q5	Q6	Q7	Q8	Q9
Kvist, T., Mäntynen, R., Turunen, H., Partanen, P., Miettinen, M., Wolf, G., and Vehviläinen-Julkunen, K. (2013)	√	√	√	√	√	√	√	√	√

## Data Availability

The articles’ data supporting this systematic review are from previously reported studies and datasets, which have been cited. The processed data are available in Table 2 and in the reference list. Further information can be requested from the corresponding author.

## Public Involvement Statement

No public involvement in any aspect of this research.

## Guidelines and Standards Statement

This manuscript was drafted against the PRISMA 2020 Statement. A complete checklist is found in Appendix A of the manuscript.

## Appendix A

**Table A1:** PRISMA 2020 Checklist.

Section and Topic	Item #	Checklist Item	Location Where Item Is Reported (Page Number)
**TITLE**			
Title	1	Identify the report as a systematic review.	1
**ABSTRACT**			
Abstract	2	See the PRISMA 2020 for Abstracts checklist.	1
**INTRODUCTION**			
Rationale	3	Describe the rationale for the review in the context of existing knowledge.	3
Objectives	4	Provide an explicit statement of the objective(s) or question(s) the review addresses.	3
**METHODS**			
Eligibility criteria	5	Specify the inclusion and exclusion criteria for the review and how studies were grouped for the syntheses.	4
Information sources	6	Specify all databases, registers, websites, organizations, reference lists, and other sources searched or consulted to identify studies. Specify the date when each source was last searched or consulted.	4
Search strategy	7	Present the full search strategies for all databases, registers, and websites, including any filters and limits used.	4
Selection process	8	Specify the methods used to decide whether a study met the inclusion criteria of the review, including how many reviewers screened each record and each report retrieved, whether they worked independently, and, if applicable, details of automation tools used in the process.	5
Data collection process	9	Specify the methods used to collect data from reports, including how many reviewers collected data from each report, whether they worked independently, any processes for obtaining or confirming data from study investigators, and, if applicable, details of automation tools used in the process.	5
Data items	10a	List and define all outcomes for which data were sought. Specify whether all results that were compatible with each outcome domain in each study were sought (e.g., for all measures, time points, analyses), and, if not, the methods used to decide which results to collect.	N/A
	10b	List and define all other variables for which data were sought (e.g., participant and intervention characteristics, funding sources). Describe any assumptions made about any missing or unclear information.	N/A
Study risk of bias assessment	11	Specify the methods used to assess risk of bias in the included studies, including details of the tool(s) used, how many reviewers assessed each study and whether they worked independently, and, if applicable, details of automation tools used in the process.	7
Effect measures	12	Specify for each outcome the effect measure(s) (e.g., risk ratio, mean difference) used in the synthesis or presentation of results.	N/A
Synthesis methods	13a	Describe the processes used to decide which studies were eligible for each synthesis (e.g., tabulating the study intervention characteristics and comparing against the planned groups for each synthesis (item #5)).	20
	13b	Describe any methods required to prepare the data for presentation or synthesis, such as handling missing summary statistics or data conversions.	N/A
	13c	Describe any methods used to tabulate or visually display results of individual studies and syntheses.	N/A
	13d	Describe any methods used to synthesize results and provide a rationale for the choice(s). If meta-analysis was performed, describe the model(s), method(s) to identify the presence and extent of statistical heterogeneity, and software package(s) used.	N/A
	13e	Describe any methods used to explore possible causes of heterogeneity among study results (e.g., subgroup analysis, meta-regression).	N/A
	13f	Describe any sensitivity analyses conducted to assess robustness of the synthesized results.	N/A
Reporting bias assessment	14	Describe any methods used to assess risk of bias due to missing results in a synthesis (arising from reporting biases).	N/A
Certainty assessment	15	Describe any methods used to assess certainty (or confidence) in the body of evidence for an outcome.	N/A
**RESULTS**			
Study selection	16a	Describe the results of the search and selection process, from the number of records identified in the search to the number of studies included in the review, ideally using a flow diagram.	6
	16b	Cite studies that might appear to meet the inclusion criteria, but which were excluded, and explain why they were excluded.	6
Study characteristics	17	Cite each included study and present its characteristics.	7
Risk of bias in studies	18	Present assessments of risk of bias for each included study.	N/A
Results of individual studies	19	For all outcomes, present, for each study: (a) summary statistics for each group (where appropriate) and (b) an effect estimate and its precision (e.g., confidence/credible interval), ideally using structured tables or plots.	N/A
Results of syntheses	20a	For each synthesis, briefly summarize the characteristics and risk of bias among contributing studies.	N/A
	20b	Present results of all statistical syntheses conducted. If meta-analysis was done, present for each the summary estimate and its precision (e.g., confidence/credible interval) and measures of statistical heterogeneity. If comparing groups, describe the direction of the effect.	N/A
	20c	Present results of all investigations of possible causes of heterogeneity among study results.	20–25
	20d	Present results of all sensitivity analyses conducted to assess the robustness of the synthesized results.	20–25
Reporting biases	21	Present assessments of risk of bias due to missing results (arising from reporting biases) for each synthesis assessed.	N/A
Certainty of evidence	22	Present assessments of certainty (or confidence) in the body of evidence for each outcome assessed.	20–25
**DISCUSSION**			
Discussion	23a	Provide a general interpretation of the results in the context of other evidence.	25–26
	23b	Discuss any limitations of the evidence included in the review.	25–26
	23c	Discuss any limitations of the review processes used.	25–26
	23d	Discuss implications of the results for practice, policy, and future research.	25–26
**OTHER INFORMATION**			
Registration and protocol	24a	Provide registration information for the review, including register name and registration number, or state that the review was not registered.	N/A
	24b	Indicate where the review protocol can be accessed or state that a protocol was not prepared.	N/A
	24c	Describe and explain any amendments to information provided at registration or in the protocol.	N/A
Support	25	Describe sources of financial or non-financial support for the review and the role of the funders or sponsors in the review.	27
Competing interests	26	Declare any competing interests of review authors.	27
Availability of data, code, and other materials	27	Report which of the following are publicly available and where they can be found: template data collection forms; data extracted from included studies; data used for all analyses; analytic code; any other materials used in the review.	31

*From:* Page MJ, McKenzie JE, Bossuyt PM, Boutron I, Hoffmann TC, Mulrow CD, et al. The PRISMA 2020 statement: an updated guideline for reporting systematic reviews. BMJ 2021;372:n71. doi: 10.1136/bmj.n71. For more information, visit: http://www.prisma-statement.org/, access on 26 March 2023.
